# Comparison of Minimal Residual Disease Detection by Multiparameter Flow Cytometry, ASO-qPCR, Droplet Digital PCR, and Deep Sequencing in Patients with Multiple Myeloma Who Underwent Autologous Stem Cell Transplantation

**DOI:** 10.3390/jcm6100091

**Published:** 2017-09-25

**Authors:** Hiroyuki Takamatsu

**Affiliations:** Hematology/Respiratory Medicine, Faculty of Medicine, Institute of Medical, Pharmaceutical and Health Sciences, Kanazawa University, 13-1 Takaramachi, Kanazawa, Ishikawa 920-8641, Japan; takamaz@staff.kanazawa-u.ac.jp; Tel.: +81-76-265-2276; Fax: +81-76-234-4252

**Keywords:** multiple myeloma, minimal residual disease, allele-specific oligonucleotide-PCR, droplet digital PCR, next-generation sequencing

## Abstract

Multiple myeloma (MM) is a hematological malignancy with a poor prognosis, characterized by clonal proliferation of plasma cells in the bone marrow (BM). Relapse due to undetected minimal residual disease (MRD) is the leading cause of death among patients with MM. This review summarizes the methods and prognostic value of MRD assessment in BM and autografts from MM patients who underwent autologous stem cell transplantation (ASCT) by multiparameter flow cytometry (MFC), allele-specific oligonucleotide real-time quantitative PCR (ASO-qPCR), droplet digital PCR (ddPCR), and next-generation sequencing (NGS)-based detection methods. MRD assessment using NGS-based approaches has clear prognostic value and better sensitivity compared to traditional methods.

## 1. Introduction

The complete response (CR) rate in multiple myeloma (MM) cases remained below 10% until the emergence of high-dose melphalan therapy with autologous stem cell transplantation (HDM-ASCT) [1,2] and novel agents such as proteasome inhibitors (PI) [3,4,5], immunomodulatory drugs (IMiDs) [6,7,8], and monoclonal antibodies [9,10,11]. These developments have increased CR rates up to 80% [12], and increasing numbers of MM patients have been able to achieve extremely deep CR wherein minimal residual disease (MRD) is not detected, even by highly sensitive methods. Hence, the International Myeloma Working Group (IMWG) recently proposed therapeutic effect assessment criteria based on MRD examination methods [13]. In this report, I review MRD detection methods that will potentially enable further stratification of CR cases in MM, according to the literature.

## 2. MRD Detection Methods

### 2.1. Multiparameter Flow Cytometry

Multiparameter flow cytometry (MFC) (four or more colors) is frequently applied in clinical practice for the detection of MRD [14,15,16,17,18,19,20,21,22], and although its sensitivity (approximately 10^−4^) is inferior to that of the allele-specific oligonucleotide-PCR (ASO-PCR) assay, there is little difference between the clinical values of these methods [15]. Previous IMWG criteria define immunophenotypic CR (iCR) as stringent CR (sCR), in which MRD is not detected by MFC [23]. Rawstron et al. used six-color MFC (CD138/CD38/CD45/CD19/CD56/CD27, with CD81/CD117 when required) to analyze bone marrow (BM) MRD in patients who underwent HDM-ASCT (*n* = 397) and those who did not (*n* = 245). They found that MRD-negative patients at day 100 after HDM-ASCT exhibited longer median progression-free survival (PFS) (28.6 vs. 15.5 months; *p* < 0.001) and median overall survival (OS) (80.6 vs. 59.0 months; *p* = 0.0183), whereas patients without HDM-ASCT who were MRD-negative after remission induction therapy only exhibited a longer PFS (10.5 vs. 7.4 months; *p* = 0.1) [14]. An analysis of the survival rates of these patients who underwent HDM-ASCT (*n* = 397) revealed that both PFS and OS were clearly stratified by MRD levels (across the 5-log MRD range (<10^−4^ to 10^−1^≤), the median PFS values were 3.1, 2.7, 1.9, 1.7 and 0.8 years, respectively (*p* < 0.001); median OS values were “not reached”, 6.8, 5.9, 4 and 1, respectively (*p* < 0.001)), and this stratification was also observed when the analysis was limited to patients who achieved CR [24].

**Figure 1:**
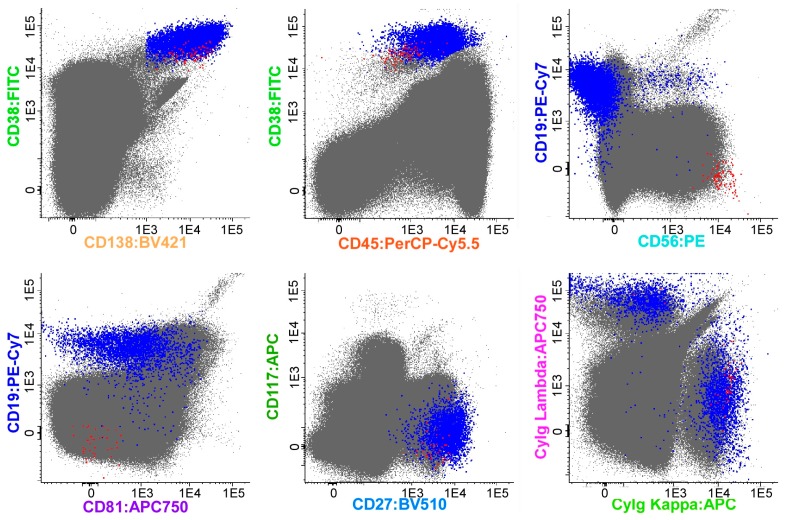
An example of the Next-Generation Flow (NGF) method based on the analysis of (merged) data files corresponding to a bone marrow (BM) sample from multiple myeloma (MM) (>10^7^ cells) with low levels of minimal residual disease (MRD), stained with the NGF-MM MRD panel. The figure shows bivariate dot plot representations in which plasma cells (PCs; blue and red dots) were gated using a conventional manual analysis strategy. Normal PCs (blue dots) display characteristic normal patterns of expression for the surface membrane markers used with a cytoplasmic (Cy) Igκ vs. CyIgλ ratio of 1.3. In contrast, clonal/aberrant PCs (red dots) can be clearly discriminated from normal PCs based on their more homogeneous phenotypic profile, the presence of myeloma-associated phenotypes (CD138hi, CD38dim, CD19-, CD81-, CD117-, and CD27dim), and a restricted pattern of expression of CyIgκ. Other non-PC BM populations are depicted as gray dots. Please note that, in this sample, PCs corresponded to 0.06% of all nucleated BM cells; in turn, aberrant PCs corresponded to 0.0007% of the whole BM cellularity, with an assay sensitivity (in the quantitative range) of <5 × 10^−6^.

According to earlier studies, the MFC technique can be applied in many patients and has advantages in terms of cost and rapidity [24,25,26], but it has not been standardized worldwide [27]. Furthermore, surface antigen patterns of myeloma cells may change from those at the time of initial diagnosis, raising the possibility of false negative results [28]. EuroFlow is therefore developing a test method called Next-Generation Flow MRD (NGF-MRD) with the aim of making it the global standard [29]. EuroFlow’s MFC is an eight-color detection method using two tubes (tube 1: CD138/CD27/CD38/CD56/CD45/CD19/CD117/CD81; tube 2: CD138/CD27/CD38/CD56/CD45/CD19/CyIgK/CyIgL) [29]. A comparative study of processing methods for hemolysis, device settings, and analysis software showed that NGF-MRD exhibited good reproducibility and detected MRD with a high sensitivity of 10^−5^–10^−6^, and its results correlated well with those of next-generation sequencing (NGS) [29]. EuroFlow is also currently developing a method to apply NGF-MRD to identify and quantify myeloma cells using the EuroFlow database and automated software. This method would allow almost complete automation of flow cytometry gating analysis. Studies have already shown that automated software results correlate well with those of expert manual analysis [29]. Figure 1 illustrates an example of the application of NGF in our department. The NGF-MRD method is expected to become widely applied worldwide soon, but its clinical value remains to be demonstrated in future clinical studies.

### 2.2. ASO-PCR

**Figure 2:**
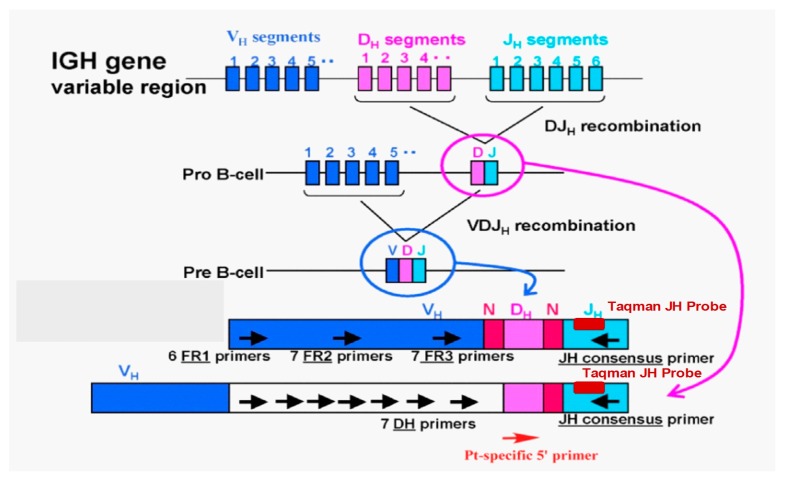
Allele-specific oligonucleotide-quantitative PCR (ASO-qPCR) method to detect minimal residual disease (MRD), and design of ASO-qPCR primers and probes.

In ASO-PCR, patient-specific primers are generated using the immunoglobulin complementarity-determining region (CDR) III, which exhibits diversity in each patient, and PCR is performed using these primers to detect MRD (Figure 2, author’s original figure) [30]. This method has an MRD detection sensitivity of 10^−4^–10^−6^ [31,32,33,34,35,36]. Puig et al. compared the results of ASO-quantitative PCR (ASO-qPCR) with those of MFC for the detection of MRD [15] and found that MRD was successfully determined by ASO-qPCR in 71 of 170 patients (42%) who achieved partial response (PR) or better. When MRD analysis was carried out in post-treatment BM in 103 patients, including 32 reported in previous studies, 54% and 46% were found to be MRD-positive by ASO-qPCR and MFC, respectively. Although a strong correlation was found between the MRD levels of ASO-qPCR and those of MFC (*r* = 0.881, *p* < 0.001), ASO-qPCR showed a greater sensitivity. Analysis of 62 patients who achieved CR showed that patients with MRD <10^−4^ had significantly better PFS (ASO-qPCR: median PFS 49 vs. 26 months, *p* = 0.001; MFC: median PFS 45 vs. 25 months, *p* = 0.001) and a significant difference in OS (ASO-qPCR: median PFS not reached vs. 60 months, *p* = 0.008; MFC: median PFS 72 vs. 45 months, *p* = 0.014). Oliva et al. presented the results of MRD evaluation by ASO-qPCR in the RV-MM-EMN-441 and RV-MM-COOP-0556 trials [37,38]. MRD assessment by ASO-qPCR was carried out after the intensification therapy or ASCT, as well as every six months until progressive disease or death during maintenance therapy. ASO-qPCR was performed according to the Euro-MRD guidelines [39]. The subjects were 105 patients who had achieved very good partial response (VGPR) or better after intensification/ASCT therapy, among whom patient-specific primers were generated for 73 patients (70%). The patients who achieved molecular CR (mCR) after intensification/ASCT therapy comprised 19/35 (54%) of those who underwent ASCT, but only 14/38 (37%) of those who did not. Of the 40 patients who had not achieved mCR after intensification/ASCT therapy, 11 (27%) achieved mCR during maintenance therapy. The impact of mCR on outcome after consolidation was as follows: median PFS was 48.8 months versus not reached in non-mCR vs. mCR patients (*p* = 0.01) (a median follow-up of 44 months). In multivariate analysis, the risk of progression/death was higher for International Staging System (ISS) stage (II/III vs. I) (hazard ratio (HR) 2.05), high-risk vs. standard-risk fluorescence in situ hybridization (FISH) (HR 2.31), age > 60 vs. ≤ 60 (HR 3.10), and non-mCR vs. mCR (HR 4.39). High-risk FISH patients with mCR had similar PFS as those with standard-risk FISH with mCR, and better PFS than non-mCR standard-risk patients (updated results were available in the poster presented at ASH 2016).

### 2.3. Droplet Digital PCR

MRD is currently being detected by ASO-qPCR, which requires calibration curves that are generated from high-quality DNA samples collected at the time of diagnosis. Droplet digital PCR (ddPCR), which does not need calibration curves, has recently been adopted for MRD measurements to avoid this problem. Its principle is as follows: the sample is divided among a large number of reaction wells, and PCR is performed for the target gene; wells that contain the target gene count as positive by PCR amplification, and those that do not count as negative. Since ddPCR involves counting of positive wells (positive rate), it offers the advantage of enabling direct and absolute quantitation without requiring comparison with a reference or standard sample [40]. Drandi et al. reported that ddPCR of immunoglobulin gene rearrangement had sensitivity, accuracy, and reproducibility comparable with those of qPCR when using BM and peripheral blood of 18 MM and 21 mantle cell lymphoma patients. However, thus far, there have been no data to predict outcomes using ddPCR in a controlled clinical setting or multi-laboratory standardization programs [41].

### 2.4. NGS

**Figure 3:**
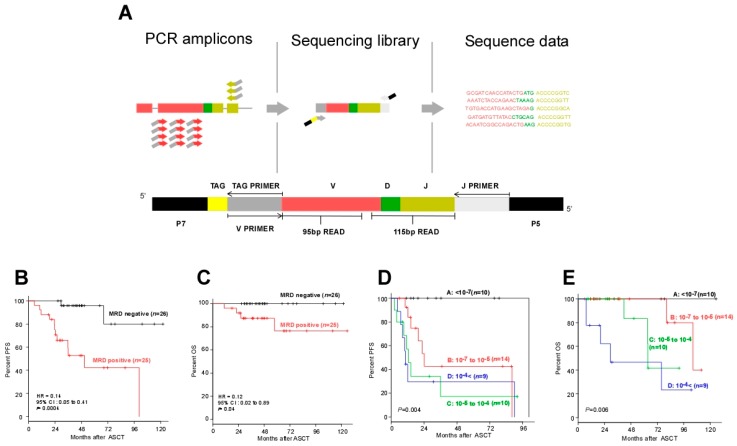
Next-generation sequencing method to detect minimal residual disease (MRD). (**A**) Schematic illustrations of LymphoSIGHT^TM^ PCR primer strategy and sequencing assay. IgH V-J, IgH D-J, and IgK sequences were amplified from genomic DNA in a two-stage PCR (the first PCR using universal primer sets, and the second PCR using J primer and TAG primer). The amplified product was sequenced to obtain a high number of reads (e.g., 10^6^ reads). The sequence reads were analyzed to determine similar sequences that form a clonotype. After clonotype determination, a standard quantitation scheme was used to calculate MRD level [43]. (**B**) Progression-free survival (PFS) and (**C**) overall survival (OS) of patients according to the MRD negativity in post-ASCT BM samples as determined by deep sequencing (threshold: 10^−6^). (**D**) PFS and (**E**) OS of the patients who did not receive post-ASCT treatment, according to MRD negativity in the autograft as determined by deep sequencing (threshold: 10^−7^).

A new method of assessing MRD has recently been developed, which combines NGS and PCR. In practical terms, patient-specific regions (the IgH-VJ/DJ and IgK regions) of DNA extracted from samples are amplified by PCR; tag sequences are added to the PCR products; and the sequences are again amplified by PCR using primers for the J and tag sequences. The patient-specific sequences of these PCR products are sequenced at least 10^6^ times at high throughput by NGS, which can detect even tiny amounts of clonal sequences in the sample. Since this method does not require the generation of patient-specific PCR primers, it is capable of cheap and rapid MRD detection at the 10^−6^ level (Figure 3A; the original figure was modified for consistency with Figure 2) [42,43,44,45,46,47].

Attal et al. applied this NGS method to evaluate MRD in BM in the IFM/DFCI 2009 clinical trial, using combination therapy with new drugs (bortezomib-lenalidomide-dexamethasone (VRD)) [48]. Of 131 MM patients who achieved CR after maintenance therapy with lenalidomide, 80 patients were MRD-negative (<10^−6^), and these patients achieved significantly better PFS than the 51 patients who were MRD-positive (three-year PFS 92% vs. 64%) [49]. At the 2016 Annual Meeting of the American Society of Hematology, MRD analysis results from clinical studies of two different combination therapies using daratumumab, an anti-CD38 monoclonal antibody, were reported: the POLLUX study of daratumumab-lenalidomide-dexamethasone (DRd) [10] and the CASTOR study of daratumumab-bortezomib-dexamethasone (DVd) [11] for relapsed/refractory MM [50]. Although the subjects were patients with relapsed/refractory MM, 12% of those who received DRd therapy and 4% of those who received DVd therapy achieved MRD negativity with a sensitivity of 10^−6^. For those who received either DRd or DVd therapy and achieved MRD negativity with a sensitivity of 10^−5^, 18-month PFS plateaued at approximately 90%, an extremely good result. At the same meeting, Zimmerman et al. also reported the results of a phase 2 trial of carfilzomib-lenalidomide-dexamethasone (KRD) plus ASCT for newly diagnosed MM patients [51]. After 18 courses of KRD therapy, 86% of patients achieved CR or better, and 74% were MRD-negative with a sensitivity of 10^−6^. Extremely good results were achieved, with three-year PFS (86%) and three-year OS (95%) for all 76 patients. For MRD-negative patients by NGS, three-year PFS and three-year OS were 89% and 96%, respectively, and those for MRD-positive patients by NGS were not presented. At our hospital, an analysis of MRD in BM after ASCT by NGS with a sensitivity of 10^−6^ found that disease progression or death will not occur in most patients even over the long term (Figure 3B,C; the reproduction of these figures is authorized) [47].

### 2.5. MRD Assessment of Autografts

The presence of MRD positivity in autografts indicates the presence of contaminating myeloma cells in the product, which will be infused back into the patient; however, the presence of MRD in the autograft could also simply show that a substantial number of myeloma cells remain in the patient’s body, and that the homogenous nature of the mobilized autograft relative to the focal nature of myeloma in BM might provide a better sample to assess MRD. The contamination hypothesis might contradict previous results [52]. Stewart et al. conducted a phase III randomized trial to study whether the enrichment of CD34^+^ autograft cells and purging of malignant plasma cells would affect PFS and OS in a cohort of MM patients receiving autografts. Despite the significant reduction in myeloma cell contamination in the autograft (median 3.1 logs), no improvement was observed in either PFS or OS. However, the application of much more effective treatments these days might change the meaning of MRD negativity in autografts.

Recently, MRD was detected in autografts used for ASCT using seven-color MFC [53]. All patients in this study underwent remission induction regimens with novel agents (carfilzomib-lenalidomide-dexamethasone (KRD), *n* = 2; bortezomib-cyclophosphamide-dexamethasone (VCD), *n* = 8; and bortezomib-thalidomide-dexamethasone (VTD), *n* = 33), and a good response was achieved (3 CR (7%) and 27 VGPR (63%) after remission induction therapy). The contamination rate in autografts with myeloma cells was extremely low in 10 of 43 patients (23%), and those patients who underwent ASCT with MRD-negative autografts exhibited significantly better PFS than those who received MRD-positive autografts for ASCT (*p* = 0.008).

The DNA in autografts of the 22 patients who achieved at least a PR after ASCT without any post-ASCT treatments was subjected to ASO-PCR. The median PFS of the eight patients with MRD-positive autografts was 18 months, whereas that of 14 patients with MRD-negative autografts was not reached at a median follow-up of 27 months (*p* = 0.012) [32]. We also performed ddPCR and NGS to assess MRD in the autografts of 18 of our patients who did not receive consolidation/maintenance therapy after ASCT and whose autografts were negative for MRD by ASO-qPCR [40]. Although a good correlation was found between the MRD levels of autografts measured by ddPCR and NGS, six patients (33%) were MRD-negative by ddPCR but MRD-positive by NGS (MRD^ddPCR^(+)^NGS^(+) (*n* = 5), MRD^ddPCR^(−)^NGS^(+) (*n* = 6), MRD^ddPCR^(−)^NGS^(−) (*n* = 7). An analysis of PFS by ddPCR and NGS found that, although in both groups PFS was better for patients who underwent ASCT with MRD-negative autografts, NGS analysis allowed a more accurate prognosis (seven MRD^ddPCR^(−)^NGS^(−) cases showed significantly better PFS than six MRD^ddPCR^(−)^NGS^(+) (median PFS 8.4 vs. 2.0 years; *p* = 0.035) [40]).

In our own hospital, an analysis of MRD in autografts in the ASCT setting by NGS with a sensitivity of 10^−7^ showed no disease progression or death in most of the MRD-negative patients, even if no further treatment was performed after ASCT, with prognosis clearly stratified by the quantitative level of MRD (Figure 3D,E; the reproduction of these figures is authorized) [47].

## 3. Effect of MRD on Prognosis: Review of Previous Meta-Analyses

There have been two recent meta-analyses of MRD in MM [54,55]. The first was a meta-analysis by Landgren et al. of four clinical trials for newly diagnosed MM patients [54], which found that MRD-negative patients showed better PFS (HR = 0.35, 95% confidence interval (CI) 0.27–0.46; *p* < 0.001) and OS (HR = 0.48, 95% CI 0.33–0.70; *p* < 0.001) than MRD-positive patients. The second was a meta-analysis by Munshi et al. of clinical studies of at least 20 newly diagnosed MM patients (controlled trials, randomized controlled trials, and cohort studies) [55]. This meta-analysis ultimately included 21 studies, and found that MRD-negative patients showed better PFS (HR = 0.41, 95% CI 0.36–0.48; *p* < 0.001) than MRD-positive patients, with similar results when the analysis was limited to CR patients (HR = 0.44, 95% CI 0.34–0.56; *p* < 0.001). MRD-negative patients also exhibited better OS (HR = 0.57, 95 CI 0.46–0.71; *p* < 0.001), with similar results when the analysis was limited to CR patients (HR = 0.47, 95% CI 0.33–0.67; *p* < 0.001). Most subjects in these two meta-analyses underwent ASCT. These results show that even in meta-analyses covering different treatments and MRD detection methods, MRD negativity is the most powerful predictor of prognosis.

## 4. Comparison of MRD Detection Methods

MFC can be applied in more than 90% of cases, and it is superior to ASO-qPCR in terms of both cost and speed. The EuroFlow protocol (NGF) achieves MRD detection with reproducibly high sensitivity (10^−5^–10^−6^) and is therefore thought to be a promising method for detecting MRD. Although ASO-qPCR is more sensitive than MFC in general, the sequencing of the CDRIII region and primer design require skill, time, and a relatively higher cost, and the success rate for primer design is only around 30–80%. Consequently, this method can be used in fewer patients [31]. ddPCR has problems with primer design similar to those of ASO-qPCR but does not require the generation of calibration curves and, if sufficient DNA is available, its sensitivity can theoretically exceed 10^−6^. Moreover, it is cheaper than NGS. ASO-qPCR and ddPCR cannot detect MRD when mutations in the CDRIII region occur [56]. On the other hand, NGS, which has recently been developed, overcomes all the above-mentioned problems with ASO-qPCR, is the most sensitive technique available, and is expected to become the main method of MRD detection. However, very few institutions are capable of carrying out NGS measurements (Table 1) [25]. In addition, because myeloma lesions are unevenly distributed in BM, a single BM aspiration test may result in a false negative result [57]. It is thus necessary to be aware of the limitations of MRD detection in BM samples, and of the need to carry out the procedure in combination with diagnostic imaging such as positron emission tomography, computed tomography, and magnetic resonance imaging.

## 5. The Way Forward

The stratification by MRD testing of myeloma patients who have achieved CR is essential for accurate prognosis in the future. Given that patients who are MRD-negative by a very sensitive detection method are unlikely to relapse or undergo tumor progression even if maintenance therapy is discontinued [44,45,47,49], such therapy should be ceased in MRD-negative patients as part of clinical studies.

## Figures and Tables

**Table 1:** Comparison of MRD assays in multiple myeloma.

	Next-Generation MFC (≥8-color)	ASO-qPCR	ASO-ddPCR	NGS
Applicability (%)	~100%	~80%	~80%	90%~
Sensitivity	10^−5^~10^−6^	10^−4^~10^−6^	10^−6^~	10^−6^~
Reproducibility	High	High	Not reported	Not reported
Diagnostic sample	Important but not mandatory	Mandatory	Mandatory	Mandatory
MRD sample	Cells	Cells, DNA	Cells, DNA	Cells, DNA
Need for patient’s specific reagent	No	Yes	Yes	No
MRD detection at clonal evolution	Possible	Impossible	Impossible	Possible
Time	3–4 h	2–3 h (follow-up), 3–4 week (target identification)	2–3 h (follow-up), 3–4 week (target identification)	≥7 d
Standardization	Ongoing (EuroFlow/IMF)	Yes (EuroMRD)	No	No
Cost per sample	~350 USD	~500 USD (follow-up), ~1500 USD at diagnosis (target identification)	~500 USD (follow-up), ~1500 USD at diagnosis (target identification)	~1000 USD

MFC, multiparameter flow cytometry; ASO-qPCR, allele-specific oligonucleotide-quantitative polymerase chain reaction; NGS, Next-generation sequencing; MRD, minimal residual disease; IMF, International Myeloma Foundation; USD, US dollars.
