# Takamatsu, H. Comparison of Minimal Residual Disease Detection by Multiparameter Flow Cytometry, ASO-qPCR, Droplet Digital PCR, and Deep Sequencing in Patients with Multiple Myeloma Who Underwent Autologous Stem Cell Transplantation. *J. Clin. Med.* 2017, *6*, 91

**DOI:** 10.3390/jcm6110106

**Published:** 2017-11-20

**Authors:** Hiroyuki Takamatsu

**Affiliations:** Hematology/Respiratory Medicine, Faculty of Medicine, Institute of Medical, Pharmaceutical and Health Sciences, Kanazawa University, 13-1 Takaramachi, Kanazawa, Ishikawa 920-8641, Japan; takamaz@staff.kanazawa-u.ac.jp; Tel.: +81-76-265-2276

The author wishes to make the following correction to this paper [1]:

In figure 3, the name “LymphoSIGHT^TM^” should be replaced with “clonoSEQ^®^”.
